# The Optical-Coenaesthetic Disproportion Hypothesis of Feeding and Eating Disorders in the Light of Neuroscience

**DOI:** 10.3389/fpsyt.2019.00630

**Published:** 2019-09-12

**Authors:** Giovanni Stanghellini, Massimo Ballerini, Milena Mancini

**Affiliations:** ^1^Department of Psychological Sciences, Health, Territory, G. d’Annunzio University of Chieti and Pescara, Chieti, Italy; ^2^Centro de estudios de fenomenología y psiquiatría - Diego Portales’ University, Santiago, Chile; ^3^Department of Mental Health, USL Centro, Florence, Italy

**Keywords:** abnormal bodily phenomena, body-for-others, bodily self-consciousness, feeding and eating disorders, multisensory integration

## Abstract

This article builds on and extends the ‘optical-coenaesthetic disproportion’ (OCDisp) hypothesis of feeding and eating disorders (FEDs) matching data obtained through clinical research with laboratory evidence from neuroscience and neuropsychological studies. The OCDisp hypothesis, developed through the assessment in clinical setting of bodily experience using the IDentity and EAting (IDEA) disorder questionnaire, argues that in persons with FED the internal perception of one’s embodied self (i.e., coenaesthesia) is deeply affected (their possibility to feel themselves is weakened or threatened by coenaesthopathic and emotional paroxysms; their bodily feelings are discontinuous over time), and as a compensation to it, these persons experience their own body as an object that is looked at by others. To FED persons, their body is principally given to them as an object ‘to be seen.’ The other’s look serves as an optical prosthesis to cope with hypo- and dis-coenaesthesia and as a device through which persons with FED can define themselves and attenuate the anxiety produced by the conflicts between being-oneself and being-for-others. After describing the OCDisp hypothesis, we will gather evidence supporting it with neuroscience studies on FED. Our focus will be on data pointing to dampened multisensory integration of interoceptive and esteroceptive signals, demonstrating a predominance of the visual afferents toward signals arising within the body. In the final part of the article, we will show consistencies but also draw distinctions between our clinical hypothesis and neuroscience-based data and hypotheses and draft a potential agenda for translational research inspired by these.

## Introduction

Abnormal bodily phenomena are among the main experiential dimensions investigated in feeding and eating disorder (FED). Generally speaking, these include abnormal body image and anomalous bodily self-consciousness.

This evidence confirms what is being established by clinical research, namely, the ‘optical-coenaesthetic disproportion’ (OCDisp) hypothesis of FED (1–3). Coenaesthesia (deriving from Greek *koiné aesthesis*, common sensorium) is the internal perception of one’s own body, the hub of somatosensations, that is of sensations coming *from within one’s body*. More in detail, it is the global experience in which all the single bodily sensations are synthesised, the crossroads of all interoceptive sensibility on which self-consciousness is grounded, including the feeling of existing, of being a self, and of being separated from the external world (4, 5). The OCDisp hypothesis argues that in persons with FED the coenaesthetic apprehension of oneself is troubled, and as a compensation to it, these persons experience their own body as an object that is looked at by others. This hypothesis was developed through the assessment in clinical setting of bodily experiences in persons with FED using the IDentity and EAting (IDEA) disorder questionnaire (6). The IDEA questionnaire assesses abnormalities in lived corporeality and personal identity. It consists of 23 items divided into four subscales: feeling oneself through the gaze of the other and defining oneself through the evaluation of the other, feeling oneself through objective measures, feeling extraneous from one’s own body, and feeling oneself through starvation. This research allowed the identification of a specific pheno-phenotype (7) that expresses a gradient of vulnerability to FED along a continuum, rising from high-risk nonclinical subjects toward the clinical population of eating disorder patients and including obese patients (8–11). In a nutshell, to these persons, their body is principally given as an object “to be seen.” The other’s look serves as an optical prosthesis to cope with hypo- and dis-coenaesthesia and as a device through which persons with FED can define themselves and attenuate the anxiety produced by the conflicts between being-oneself and being-for- others. This article aims to match clinical data supporting the OCDisp hypothesis with evidence taken from laboratory (neuroscience and neuropsychological) studies on FED and, building on and extending these, draft a potential agenda for translational research inspired by the OCDisp hypothesis.

Both body image and bodily self-consciousness derive from complex integrative processes between different perceptual domains, which mainly include bodily signals coming from within the body (interoception) and esteroceptive stimuli (among which visual inputs are particularly relevant). Evidence obtained through research in the neurosciences suggests that body image and bodily self- consciousness are impaired in FED persons. In particular, dampened multisensory integration of interoceptive and esteroceptive signals, demonstrating predominance of the visual afferents toward signals arising from within the body, plays a major role in abnormal body and self experiences in persons with FED (12–22).

## The Optical-Coenaesthetic Disproportion Hypothesis of FED

There are theoretical as well as clinical reasons to consider abnormal eating behaviours as epiphenomena of a more profound disorder of lived corporeality. Under normal conditions, bodily experience is combination of the way I feel myself from a first-person perspective and the way I experience myself through other sense modalities, one of the most important of which is sight.

Through sight, I see myself from a third-person perspective. Yet sight is also involved when I also experience myself as an object seen by others. This is a peculiar feature of FED psychopathology. There is empirical evidence that FED persons feel extraneous from their own body (6, 8, 11, 23); their possibility to feel themselves is weakened or threatened by coenaesthopathic and emotional paroxysms; their bodily feelings are discontinuous over time (23). Since their experience of their body from within is flawed or inconsistent, they cope with this by apprehending their body from without through the other’s gaze. They experience their body as an object being looked at by another, rather than coenaesthetically or from a first-person perspective (24). What they seem to lack is the coenaesthetic apprehension of their own body as the more primitive and basic form of self-awareness (25). The way they feel looked at by the others is the principal mode to feel themselves and define their identity (6). Their body is principally given to them as an object “to be seen.” We called this peculiar way of apprehending one’s own body after Sartre (26) *body-for-others*—a body exposed and subjected to the other’s gaze and thus reduced to its appearance.

As the first-person apprehension of one’s body is based on coenaesthesia, whereas the third-person one is based on the sense of sight, we may call the dynamic balance between the apprehension of one’s body through coenaesthesia and through the other’s look the *optical-coenaesthetic proportion*—a prerequisite for constructing a safe and dependable sense of bodily self and personal identity. Under normal conditions, the constitution of our own body, and consequently of our own self and identity, depends on the dialectic integration between these two perspectives. In persons with FED, this dialectics breaks down. Particularly relevant to understanding a person with FED is to envision in the other’s look a kind of visual prosthesis that helps him/her feel his/her own body. Feeling one’s body as an object being looked at by another has a twofold effect: it makes FED people feel embarrassment and repulsion for their own body, but it also helps them recover a sense of selfhood, “unity,” and “condensation” (27). This phenomenon is epitomised by the following micro-narratives: “The way I feel depends on the way I feel looked at by the others,” “Sometimes I focalise myself through the gaze of the others,” “For me it’s very important to see myself through the eyes of the others,” “Even if I think that the way the others evaluate me is wrong, I can’t do without it” (6).

## Bodily Self-Consciousness: Neural Bases and Controversies

Several—and somehow controversial—constructs are available in neuroscience literature to depict the experience of the body, such as body image (BI) and bodily self-consciousness (BSC). Body image generally refers to inputs from the body (28). We endorse in this article the definition of BI (29). It encompasses a perceptual (body perception), an affective, and a cognitive domain (13). Each of these domains has distinct brain localisations: perceptual—posterior parietal cortex; affective—amygdala, insula and the prefrontal cortex; cognitive—parietal regions (30, 31). A more nuanced localisation of the cortical areas specifically activated especially by visual body perception includes the extrastriate body area (the whole body and its components) and the fusiform body area (the configurational picturing of the whole body), as well cortical areas activated by face perception such as the fusiform face area, the occipital face area, and the posterior superior temporal sulcus (32). These systems constitute the detection network (33), while many other cortical regions are implied in the subsequent processing of the expressive, emotional, semantic, and cognitive features connected to body, face, and gaze.

A large part of research on the perceptual domain of BI is about its visual component (13, 34). Yet BI can be seen as the integration of egocentric signals coming from the body and allocentric inputs coming from the environment mediated by other sensory domains.

Also, online BI can be separated from offline BI (35). Online BI derives from egocentric signals coming from within the body itself, whereas offline BI is a more stable representation of the body, which may be assimilated to the long-term, memory stored BI (35, 36), comprehensive also of behaviours, attitudes, and body pertaining values.

Body image thus results from very complex integrative processes and dynamic interplay: perception and action, different perceptual domains (e.g., bodily signals coming from within the body and visual stimuli), egocentric and allocentric afferents, short- and long-term information, and online and offline inputs. To depict the integrative processing of egocentric online afferents and allocentric long-term stored information, the Bayesian error-prediction computational model is widely accepted (37): a reference-framework of body representation, memory-stored, aligns the incoming stimuli from different sensory systems according to the principles of predictive coding and free energy—where the first is the tendency to reduce the differences between predictions and incoming afferents (38), and the latter is the tendency to resist to possible disorder deriving from inconsistent matching (39). Notably, the incoming multisensory bodily signals represent the basic feature of selfhood. In this sense, the primary experience of the body is constitutive of self-consciousness; this implicit, prereflexive background is also designated as BSC.

Bodily self-consciousness is the multisensory integration of the afferents coming from within and from outside the body (esteroceptive inputs), including a) proprioceptive and vestibular signals indicating the position of the whole body and its components in the space; b) auditive and visual data concerning body shape and structure; and c) multisensory integration of bodily and esteroceptive stimuli, necessary to the development of peripersonal space, i.e., the space immediately surrounding our body, with diverse extension in correspondence of the trunk, face, and the arms. Further information contributes to the constitutive process of BSC: d) interoceptive afference regarding the physiological (homeostatic) condition of body organs and bodily functions (e.g., hunger or satiety) and e) the sense of agency regarding body actions (37).

The multisensory information is integrated in bimodal or multimodal neurons located mainly in parietal cortex, activated by afferents (visuotactile or auditive, proprioceptive, and kinestesic modalities) coming from primary sensory areas; the cortical areas involved in BSC include the premotor cortex, posterior parietal cortex, and particularly the intraparietal sulcus and the temporoparietal junction, the latter with functional connectivity with the right insula and the supplementary motor area (40). The insula has been recognised as a central hub for interoceptive signals (41). The BSC accounts for the basic features of selfhood: 1) self-identification, the immediate sense of ownership regarding the whole body and its components; 2) self-location, the sense of bodily position in the environmental space; 3) self-perspective, intended as the point of reference of our perceptual engagement in the world; 4) self-demarcation, since the peripersonal space draws the effectively lived self-boundaries; 5) self-agency, or the sense to be the operator of any motility engagement in the world (42–44).

A striking BSC characteristic is its plasticity (45). Long-lasting multisensory stimulation manipulating the spatiotemporal coherence of bodily signals alters BSC by reshaping peripersonal boundaries and inducing BSC for noncorporeal objects. The best known experimental paradigm to explore BSC plasticity is the rubber-hand illusion (RHI) where the probands’ arm is hidden in a special machinery that consents to see only an artificial rubber hand. In RHI, there is a predominance of visual sensory afferents that reshape bodily (and subcomponents) limits and ownership. Prolonged tactile stimulation of the probands’ real arm and—at the same time—the artificial hand may induce the illusion of ownership toward the fake hand (46).

## Neurological Underpinnings of FED: Integration and Disintegration of Interoceptive and Esteroceptive Inputs

A relatively small number of studies investigated body sensory networks in FED patients. The results of these studies can be summed up as follows: FED persons show diminished interoceptive/somatosensory signals, overreliance on visual afferents, and dampened multisensory integration of visual and interoceptive/somatosensory signals.

*Diminished interoception*. The interoceptive component of BSC is based on egocentric signals coming from the body (47). Interoception includes the physiological state of the entire body, concerning autonomic nervous system information about the condition of the body (e.g., taste, smell, hunger, thirst, and visceral sensation). Interoception is regarded as an essential component of subjective emotional experience in key theories of emotion including Damasio’s (48, 49) somatic marker hypothesis that argues that perceiving changes in the bodily state (autonomic bodily signals) are the basis of our emotional experiences. People who have reduced interoceptive sensitivity have a correspondingly reduced experience of emotions. Patients with FED display impairment in haptic, proprioceptive, and interoceptive sensitivity demonstrating altered bottom-up processing of bodily signals (13, 33). Healthy subjects with BI disturbances (body dissatisfaction and uneasiness and BI avoidance) display the tendency to incorrectly estimate their body measures when exposed to a virtual reality paradigm, for instance, when they evaluate virtual avatars with varying body massindexes (50). These impairments resulted evident in allocentric, third-person perspective (viewing the avatar body as they were other people who observed them).

*Overreliance on visual afferents*. Increased proneness to RHI in healthy people correlates with reduced interoceptive accuracy (51), demonstrating a predominance of the visual afferents toward signals arising within the body and, consequently, a greater BSC malleability. People with reduced interoceptive accuracy display a heightened tendency to self-objectivisation (the tendency to rely for self-identity upon mere body appearance), experiencing their own body from a third-person perspective (52). Increased proneness to RHI is also documented in anorexic (AN) patients (45) and in a group of FED patients (47); these patients display a more evident proprioceptive drift (a misconception of the position of the real finger) and a higher embodiment score (extension of ownership toward the fake arm). These findings put in evidence an overreliance to visual information that become dominant with respect to interoceptive signals. Zopf et al. (53) obtained similar findings in a group of AN patients, inversely related to the length of illness. Finally, Keizer et al. (54) documented that in AN patients exposure to virtual reality experiments reduces the tendency to overestimate their body size, suggesting a reshaping of BI induced by a modification of the BSC.

*Dampened sensory integration*. Some evidence for a dampened multisensory integration of visual and interoceptive/somatosensory signals has emerged from the functional magnetic resonance imaging study of brain resting states measuring functional connectivity of brain networks. Divergent procedures to acquire the signal (i.e., independent component analysis, box-seed investigation of predefined regions of interest, graph analysis), small patients sampling (with possible interindividual distribution), and clinical variability (including the co-occurrence of psychiatric comorbidities) may account for inconsistent findings. Nonetheless, some interesting evidences has emerged.

Underweight AN patients display decreased connectivity in the ventral visual network (left occipitotemporal junction, a region implied in short-term memory persistence of visual stimuli and body perception) (55). Recovered AN patients show a significant hypoconnectivity in the right middle frontal gyrus, involved in spatial working memory and also implied in the updating of spatial information. In both groups, hyperconnectivity in somatosensory network including the premotor areas was demonstrated. Impaired visuosomatosensory signals integration may be the root of altered body experience in AN patients. Decreased connectivity in the thalamoinsular subnetwork is suggestive of impaired interoception in AN patients (56).

Similarly, Phillipou et al. (57) in a sample of underweight AN patients highlighted hypoconnectivity between primary somatosensory and both motor cortical areas as secondary associative visual cortices. Lavagnino et al. (58) found in a small sample of unmedicated bulimic women decreased connectivity within the somatosensensory network and between the paracentral lobule and the right middle occipital gyrus, the right cuneus, and the posterior cingulate cortex (PCC), the latter invoked for self-specificity processing. This finding may substantiate the experience of extraneity referred by patients toward own’s body. Defective connection between the paracentral lobule and the EBA may be referred to a multisensory defective integration (also, this finding displayed a negative correlation with interoceptive accuracy). Scaife et al. (59) found a set of decreased connectivity in recovered and restrictive AN patients, within the lateral visual area, the right temporal/temporal-occipital fusiform cortex (an area associated with face and body recognition), and finally in several cortical regions associated with interoceptive and somatosensory functions, documenting the impairment of body processing. The presence of brain networks anomalies in recovered patients can be considered a possible trait character or, alternatively, a sort of scar depending on the previous state of malnourishment.

## The Allocentric Lock Hypothesis of FED

Taken together, all these laboratory findings highlight the anomalous processing of bodily sensory afferents, the predominance of visual inputs, and the defective multisensory integration of the stimuli coming from within the body with the esteroceptive (visual) perceptions, indicating a defective constitution of the BSC in persons with FED. All this is nicely encapsulated in the allocentric lock hypothesis (ALH). There are several analogies between this and the OCDisp hypothesis, in particular the emphasis on the role of dampened multisensory integration of interoceptive and esteroceptive signals in the pathogenesis of anomalous bodily experiences in FED persons. Comparing these two hypotheses can help to amalgamate the clinical evidence gathered by the OCDisp hypothesis with data taken from the neurosciences captured by the ALH. Indeed, the ALH argues that FED symptoms are the outcome of a primary distorted experience of the body (19, 60) and more precisely consequences of the impairment of the process of integration between the egocentric experience of the body and the allocentric representation of it. If the process is impaired, the egocentric sensory inputs are no more able to update the contents of the allocentric representation of the body. The outcome of this impairment is that the subject is locked to the allocentric representation of his body, which primes the processing of any further body-related experience.

It is argued that there are two frames within which we gain access to our body: the egocentric and the allocentric. The egocentric frame (body as reference of first-person experience) is perceptive/experiential since it has its primary source on somatoperceptions and other sensory inputs such as tactile stimuli. It is field-open and unceasingly online since its inputs are constantly updated by new inputs. Thus, the kind of bodily experience it conveys is discontinuous over time. These are engrams of the present state of the body taking place within short-term memory processes. The allocentric frame (body as object in the physical world) is representational since it has its primary contents in somatorepresentations—abstract knowledge, beliefs, and attitudes related to body as an object of third-person experience. It is offline and in principle continuous over time if its representations are not updated by inputs coming from the egocentric frame. It is encoded in long-term memory and located in the hippocampus and surrounding medial temporal lobe.

The egocentric frame corresponds to the unmediated, first-person experience of oneself as a spatiotemporal embodied agent—neither a representation of one’s body, nor its perception as an external entity. It is the body experienced from within—how the body feels. The allocentric frame corresponds with the body seen from a third-person perspective as an entity existing in the outside world or perceived from without (e.g., when I look at myself in a mirror or remember my visual image). Sight is the sense modality through which I perceive my body from without as an object-body—how the body *looks*.

Under normal conditions, the egocentric experience of how our body feels is matched by an allocentric one. The interaction between egocentric and allocentric frames corresponds to the interaction between long- and short-term memory processes. Specifically, long-term memory involves the generation of allocentric representations, whereas short-term memory contains egocentric experiences driven by perception or by long-term memory. On one side, an egocentric experience of the body can influence an allocentric representation stored in long-term memory. On the other side, an allocentric representation of the body retrieved from long-term memory can influence egocentric sensory inputs, including body dimensions. If, for some reasons, the first process is impaired, the egocentric short-term sensory inputs are no more able to update the contents of the allocentric long-term representation of the body: the subject is locked to the allocentric representation of his body. This is what supposedly happens in persons with FED: their long-term offline allocentric body representation amounts to a negative self-image, driven by these patients’ extreme sensibility to what they experience as the others’ disapproving gaze and remarks, often eliciting in them feelings of shame and disgust related to their own body. This undesirable body representation is not updated by contrasting egocentric inputs driven by short-term online body perception; thus, the processing of any further body-related experience is impaired. The impossibility of updating the negative allocentric representation of the body locks the patient into an unsatisfying body.

## Conclusions

Both clinical and laboratory findings show impaired BSC in persons with FED. This helps conceptualizing FED as disorders of embodiment rather than as merely disorders of eating and feeding behaviour. Neuroscientific data and clinical evidence confirm each other in drawing attention to three anomalous features of bodily self-experience: diminished or impaired interoception/coenaesthesia, increased esteroception *via* visual inputs, and abnormal integration between these two sources of bodily self-experience. In detail:

1. There is a general agreement in highlighting hypo- and dis-interoception/coenaesthesia in FED. Incidentally, coenaesthesia does not account for all BSC “because this entails a variegated network of integrated sensory modalities including egocentric/allocentric, online/offline, short-term/long-term stimuli. In the light of neuroscience studies, coenaesthesia (a key concept in phenomenological studies) can be redefined as egocentric, online, short-term somatoperception; this is diminished in persons with FED.
2. FED persons also show predominance of allocentric sources of BSC, namely, inputs from the visual domain. A peculiar source of allocentric visual inputs particularly relevant in establishing BSC in FED people is the body-for-others domain. This can be seen as an esteroceptive/allocentric/visual self prosthesis, compensating diminished coenaesthesia/interoception, but locking body perception in FED people to the gaze and evaluation of the others. The body-for-others is a candidate further dimension of BSC. There is clinical evidence for this, but laboratory (neuroscience and neuropsychological) evidence is not yet available.
3. Finally, BSC in FED people is also impaired in terms of anomalous integration between different frames of bodily self-experience (interoception/esteroception, egocentric/allocentric, coenaesthetic/visual, etc.). After our clinical research, we called this the OCDisp hypothesis. Further clinical and laboratory studies are needed to confirm this finding and expand this hypothesis.

## Author Contributions

GS contributed to the conceptualization of the optical-coenaesthetic disproportion hypothesis and manuscript draft. MB contributed to the narrative review of neuroscientific literature and revision of manuscript draft. MM contributed to the acquisition of neuroscientific literature, revision of manuscript draft and project management.

## Funding

The publication fee is borne by research funds of the Department of Psychological Sciences, Health and Territory, G. d’Annunzio University of Chieti-Pescara.

## Conflict of Interest Statement

The authors declare that the research was conducted in the absence of any commercial or financial relationships that could be construed as a potential conflict of interest.

The reviewer CM declared a shared affiliation, with no collaboration, with several of the authors GS and MM to the handling editor.
